# Medical Association of Georgia

**Published:** 1886-05-20

**Authors:** 


### MEDICAL ASSOCIATION OF GEORGIA.

The editors of this journal were both present at the late meeting of the Medical Association of Georgia, at Augusta, on the 21st instant. The attendance was good, and more than usual interest manifested in the meeting by the members present.

The Association met at Clara Hall and was called to order by Dr. Eugene Foster, the retiring President.

Prayer was made by Rev. Chauncy Williams, and an address of welcome by J. R. Lamar, Esq. An address of welcome was also made in behalf of the municipal authorities, by C. H. Cohen, Esq.

These addresses were responded to in appropriate terms by Dr. E. Richardson, of Cedar Town.

Dr. Eugene Foster, after thanks for the honor of the position he had held as President for the past year, introduced the president elect, Dr. R. J. Nunn, of Savannah, who assumed the duties of the presidency. His opening address was in all respects an able one, and contained many valuable suggestions to the Association. Among other matters he alluded to the extraordinary mortality of the members during the past year, amounting to eleven deaths in a membership of only 264.

The President called attention to "a decrease in interest in the Society through the State, and urged a larger membership and more united effort to make the Association a thoroughly representative State organiztion. The President enveighed strongly against "puffs" in secular newspapers and the ignoring of the ethics of the profession by doctors, who give certificates to patent medicines and secret prescriptions. He advocated a series of prizes for original researches in the profession to be known as the President's prizes, and for which each President should contribute $100 per annum. If this proposition is received with favor by the Society, he authorized the Committee on Prizes to draw on him for $100."

Dr. Nunn touched upon sanitary measures and the economy of public health. "*Salus populi suprema est lex.*" He referred to the indifference of the State to Medical education. Laws designed to protect the community against quackery are imperfect and rarely enforced Twenty-nine States have passed laws of this character. In five of the States diplomas are not recognized as sufficient evidence of competency. These are Alabama, Arkansas, Mississippi, North Carolina and Virginia. They require all practitioners to go before an examining board. Dr. Nunn favored a high standard of education. Medicine not a science. Occupies the border-land between the sciences and arts. Theory in medicine is the parent of quackery. The quack is always ready to give a plausible reason for everything that happens. It requires a large experience to elevate a physician to the plane where he can say: "I don't know."

The closer we keep to facts and the further from theory, the better physicians we are.

It is fashionable to decry our home colleges and to exalt those of Europe. This is a great mistake. The best place to study medicine is where the physician will practice among the types of diseases he will have to treat.

The educational facilities of America are quite equal to any in the world. Original thinkers are as numerous in our own country as elsewhere. Brilliancy and dash in execution are the birthright of Americans; coolness and self-reliance are characteristic traits of American physicians.

This is especially the case with the country doctor, who must depend upon his own resources and meet grave responsibilities alone and unaided. We may not forget that America is the home of a McDowell, a Sims, a Mott, a Gross, and like eminent men in the profession. The birthland of anesthesia, ovariotomy, and, we may say, of gynecology, cannot be wanting in originaility.

Where people are most ignorant and human life is of least value, there we will find medical education at a low ebb; and on the other hand when a country is high in the social scale, and where legislators are more refined and advanced there a higher estimate is placed upon medical education and better laws for its promotion are adopted.

In Europe governments are far more exacting in the requirements of education, and more liberal in endowing institutions.

In this country, though educational institutions are in the hands of the profession, medical sectarianism has so divided our ranks that little can be expected from this source. Little is hoped from State legislatures, and general legislation, at least for many years to come. All the schools opposing regular medicine are at war with all efforts in this direction. To these the politician will cater. The peculiarities of our political system are in the way, and partizanship too often disregards the dictates of sense and of wisdom.

No system of medical education is complete that does not keep in view the great principle of *universality of medicine.* A diploma should be evidence of medical eompetency everywhere and in all countries. If our medical education is only local, then an international medical congress is a delusion.

Dr. Nunn advocated the founding of a National degree by the Government of the United States, and the appointment by the General Government of an Examining Board for the United States. We would thus establish a degree requiring the best possible medical education. To the physician, the possession of such a degree would be ardently sought, and the man who held it would have a world-wide recognition. The Doctor's remarks upon this point were very interesting, and the idea will probably become a subject for the consideration of the International Medical Congress.

We have only touched upon some of the points in Dr. Nunn's very interesting address, as our space does not permit a full report. The address was well received, and referred to a special committee for consideration and report thereon.

On Thursday, the 22d—the second day of the Association—reports of Sections were called for, and many valuable papers were presented. There were also a number of interesting voluntary papers submitted ; among these, one on the subject of Temperance, by Dr. J. P. Logan, which looked to an expression of the society upon the possibility of dispensing with alcoholic liquors. Considerable discussion ensued, participated in by Drs. Bizzell, Taliaferro, Powell, Campbell, and others. A proposition to appoint a committee to investigate and report at a future meeting was laid on the table—a majority holding to the view that it would jeopardize the harmony of the body to attempt any deliverance upon this question at the present time. There were also voluntary papers by Drs. Foster, Hammell, Elkins, Wright, Mulligan, Gaither, Doughty, Borcheim, and others.

Dr. T. S. Powell, on the bill presented to the last legislature on Practical Anatomy, stated that he had made diligent effort to secure the passage of the bill, which failed, however, by only a single vote. On motion, the same committee was continued, with instructions to renew their efforts to obtain the enactment of the law.

Dr. Beasley offered a resolution that the afternoon of the second day of the next and each subsequent meeting of the Association, hereafter, be set apart for omnibus discussion. Adopted.

Dr. Love reported, as the fraternal delegate to the Alabama State Medical Association, and gave a full account of the plan and workings of that Association—which was ordered to be printed.

The following delegates were appointed to the American Medical Association, which meets in St. Louis on May 4: H. F. Campbell, T. S. Powell, J. P. Logan, W. F. Westmoreland, J. A. Gray, G. W. Mulligan, E. C. Goodrich, A. W. Calhoun, T. F. Walker, J. F. Alexander, M. P. Deadwyler, W. F. Holt, C. H. Hall, S. B Hawkins, V. H. Taliaferro, A. G. Whitehead, T. O. Powell, A. W. Griggs, R. H. Taylor, R Battey, E. H. Richardson, S. C. Benedict, J. W. Bailey, W. B. Wells, E. W. Lane, R. C. Word, G. H. Noble, E. L. Connally, J. McF. Gaston, E. Foster, G. C. Hammell, W. S. Elkin.

The Committee on Publication recommended a return to the former method of publishing the Transactions of the Association, *a recommendation which was passed without a dissenting vote!*

The committees and appointments from the several Districts we hope to give in our next.

A paper by Dr. McHatton, on Hemorrhagic Malarial Fever, caused a lively and interesting discussion, in which Drs. DeSaussure Ford, Whitehead, Baxley, Sample, Lane, Hit, and others took part. A statement was made that if the parties who participated in this discussion would write out their remarks they would be published in the Transactions.

The officers elect for the next year are as follows: President, T. O. Powell of Milledgeville; First Vice-President, G. W. Mulligan, of Washington; Second Vice President, E. H. Richardson, of Cedartown; Censor, long term, S. B. Hawkins, of Americus; Censor, short term, Robert Battey, of Rome; Orator, W. A. O. Daniel.

Atlanta was appointed for the next meeting, to take place on the third Wednesday in April, 1887. The following is the Committee of Arrangements for Atlanta, to wit: Drs. J. F. Alexander, J. C. Baird, J. S. Todd, W. P. Nicolson, A. W. Calhoun, E. L. Connally, W. S. Elkin, J. C. Olmstead, V. O. Hardon, J. A. Gray.

On Wednesday evening our Managing Editor, with a number of medical brethren, had the honor of an invitation to tea at the elegant residence of Dr. Eugene Foster, who, with his accomplished lady, entertained us with true Georgia hospitality. The supper was all that the most epicurean palate could wish, and the happy and cordial manner of the reception given us will be long remembered. After a season, we repaired, with Dr. Foster and his lady, to the residence of Dr. Henry F. Campbell, whose hospitable mansion was, on the same night between the hours of 8 and 12 o'clock, thrown open to all the members of the Association. Dr. Campbell, in company with his accomplished daughter, Mrs. Doughty, and dressed after the old Virginia custom, in his reception coat, received his guests in that peculiarly affable and cordial manner for which he is noted. Everybody was made welcome, and the large double parlors, elegant in arrangement and gaily lighted, and with a table of delicacies and refreshments hard by, presented a scene of social handshaking and enjoyment exceedingly refreshing and acceptable to medical men coming up from the cares and hardships of professional life.

We should not omit to mention in this connection, an elegant dinner and delightful social hour enjoyed by our Senior Editor, with a number of medical brethren, at Dr. Eugene Foster's, at noon of the second day of the Association, which was a most enjoyable occasion.

THE BANQUET given at the Planters' Hotel by the citizens and physicians was a most brilliant affair. The tables groaned under the weight of choice refreshments ; toasts were drank and responded to, and much fun and social enjoyment prevailed until late at night.

In conclusion, we are constrained to express in strong terms our admiration of Augusta and her people, her manufactures, her canal and water-power, her "Sandhills," with its beautiful and charming suburban villas ; her Greene street, the prettiest in all the South, and other attractions, which, taken together, make Augusta one of the most beautiful and delightful cities in the Union.

In addition to the parties already referred to as contributing to the pleasures of our visit, we are pleased to acknowledge pleasant social attentions and courtesies from Prof. DeSaussure Ford, the eminent son of the honored and lamented Prof. Lewis D. Ford, and Col. E. R. Dorsey, the able and genial Railroad official of that city. W.

